# Non-Essential Trace Elements Dietary Exposure in French Polynesia: Intake Assessment, Nail Bio Monitoring and Thyroid Cancer Risk

**DOI:** 10.31557/APJCP.2019.20.2.355

**Published:** 2019

**Authors:** Monia Zidane, Yen Ren, Constance Xhaard, Axelle Leufroy, Suzanne Côte, Eric Dewailly, Laurent Noël, Thierry Guérin, Patrick Bouisset, Solène Bernagout, John Paaoafaite, Jacques Iltis, Marc Taquet, Eric Suhas, Frédérique Rachédi, Jean Louis Boissin, Joseph Sebbag, Larrys Shan, Frédérique Bost-Beze, Patrice Petitdidier, Carole Rubino, Jacques Gardon, Florent de Vathaire

**Affiliations:** 1 *Radiation Epidemiology Group, Centre for Research in Epidemiology and Population Health (CESP), UMR 1018 Inserm, *; 2 *Gustave Roussy, Villejuif,*; 3 *Faculty of Medicine, University Paris Sud 11, Le Kremlin-Bicêtre,*; 4 *Université de Paris-Est, Anses, Laboratory for food safety, F94700 Maisons-Alfort,*; 8 *Institute of Research for Development (IRD), Laboratoires d’études rurales, Montpellier, *; 15 * Institut de Recherche pour le Développement (IRD), Hydrosciences (HSM), Montpellier, France, *; 5 *Area of population health and optimal health practices, CHU de Québec Research Center, Québec, Canada,*; 6 *Laboratoire d’Étude et de Surveillance de l’Environnement, Institut de Radioprotection et de Sécurité Nucléaire,*; 7 *Institute of Research for Development (IRD), *; 9 *Non-Communicable Diseases Unit, Oceanian Islands Ecosystems-UMR 241, Louis Malardé Institute, *; 10 *Endocinology Unit, Territorial Hospital Taaone,*; 11 *IPRAME, *; 12 *Paofai Clinic,*; 13 *Endocrinologist,*; 14 *Laboratory of Anatomy and Cytopathology, Territorial Hospital Taaone, Papeete, French Polynesia. *

**Keywords:** Thyroid cancer, mercury, cadmium, lead, arsenic, diet, fingernail, case, control study

## Abstract

**Background:**

In French Polynesia, thyroid cancer mortality and incidence is reported to be the highest in the world. Excessive levels of non-essential trace elements (nETE) in the body are associated with several types of cancer.

**Objective:**

The present study aims to provide quantitative information on food contamination by mercury (Hg), lead (Pb), arsenic (As) and cadmium (Cd) in French Polynesia and its potential correlation with measurements performed in fingernails of Polynesians, and then to investigate the potential association between these nETE and different thyroid cancer risks.

**Methods:**

The study population included 229 interviewed cases and 373 interviewed controls We performed a descriptive analysis of Polynesian food and examined the association between thyroid cancer risk and daily intake levels of nETE and with fingernail nETE levels.

**Results:**

Hg contamination was mainly present in sea products, Pb contamination was present in almost all samples, Cd was detectable in starchy food and As was detectable in all sea products. No patient exceeded dietary contamination WHO limits for Pb, 2 participants exceeded it for Hg and 3 individuals (0.5%) for cadmium. In fingernail clippings, the most detectable pollutant was Pb (553 participants), then Hg (543 participants) then Cd (only in 130 participants). Thyroid cancer risk was increased more than 4 times by Pb daily intake in patients with a history of cancer in first-degree relatives than in ones without (p for interaction =0.01), and 2 times more in women with more than 3 pregnancies than in those with none or less (p for interaction =0.005); it was also increased following As intake by more than 30% in patients with a history of cancer in first-degree relatives than in ones without (p for interaction =0.05).

**Conclusion:**

Locally produced foods are not a source of nETE exposure in French Polynesia. Dieatry nETE exposure and fingernail nETE concentration are not associated to differentiated thyroid cancer risk. No correlation found between nETE dietary exposure and fingernail nETE concentration.

## Introduction

Thyroid cancer is the most common malignancy of the endocrine system (Ron et al., 2006). Even though it accounts for less than 2% of all cancers diagnosed worldwide, it has become the fifth most common cancer in women (Pellegriti et al., 2013). Thyroid cancer incidence varies worldwide, implicating different factors such as heritability and genetic factors, which have very strong effect in thyroid cancer occurrence (Czene et al., 2002). Environmental factors are also strongly implicated in the aetiology of this cancer. Except for ionising radiation, risk factors for thyroid cancer are not well known (Ron et al., 2012). In French Polynesia, thyroid cancer mortality and incidence (Vu et al., 2000; de Vathaire et al., 2010) was reported to be the highest in the world, in particular among natives of French Polynesia (Gleize et al., 2000).

As reported by epidemiological, clinical, and experimental studies, the development of thyroid goitres and cancer could be influenced by the environmental excess, deficiency, or imbalance of more than twenty chemical elements (Zaichick et al., 1995; Sin et al., 1992; Kvíčala et al., 1992).

Non-essential trace elements (nETE), like cadmium (Cd), lead (Pb), mercury (Hg) and arsenic (As), have no beneficial function in the body. However, excessive exposure to nETE may cause adverse health effects such as neurologic disease, renal disease, cardiovascular disease, and cancer (Fewtrell et al., 2004, Järup et al., 2003). The International Agency for Research on Cancer (IARC) classified Cd and As as human carcinogens and Pb as a suspected human carcinogen. Hg also is considered to be a possible human carcinogen (IARC, 1993) Excessive levels of nETE in the body are associated with several types of cancer such as breast cancer (Nagata et al., 2013), prostate cancer (Guzel et al., 2012) , and brain cancer (Al-Saleh et al., 2001). A relationship between low-to-moderate Cd exposure and mortality had been associated with lung and pancreatic cancer (García-Esquinas et al., 2014). Besides, several epidemiologic studies showed that As exposure is positively associated with different cancers risk such: kidneys and bladder cancer (Saint-Jacques et al., 2014; Smith et al., 1992), lung cancer (Sawada et al., 2013; Melak et al., 2014) and skin cancer (Karagas et al, 2015).In addition, Pb levels are also associated with breast cancer (Alatise et al., 2010), and liver cancer and leukaemia are linked to Hg levels (Kinjo et al., 1996).

Thyroid tissue could accumulate both toxic elements (like Hg or Cd) and essential elements (like selenium (Se) and iodine (I)); the element concentrations were much higher than those found in the other body tissues (Zaichick et al., 1995; Chung et al., 2016).

Some trace elements including Se and zinc (Zn) have been known to have anti-carcinogenic effects (Prasad et al., 2009; Hammouda et al., 2009) These findings show that the carcinogenesis can be a result of the direct influence of the element on thyroid tissue.

Numerous attempts have been made to associate nETE exposure, which dietary way is one of the most important, with thyroid diseases, including thyroid cancer risk (Tiran et al., 1995; Falnoga et al., 2000; Falnoga et al., 2006; Zagrodzki et al., 2010).

In French Polynesia, changes in lifestyle and the multiplicity of environment pollution sources affected locally produced foods such sea products (Richardson et al., 2015) which are frequently consumed by the Polynesian population. An assessment of this type of exposure is needed to determine the dietary intake level of nETE among Polynesians and to study its association with thyroid cancer risk.

However, monitoring nETE exposure requires quantification by a marker concentration dosage. According to many studies, nails could be used as nETE markers since they are easily reachable and reflect relatively long-term exposure (nearly 1 year), (He et al., 2011; Momen et al., 2015). This dosage allows the actual exposure to be evaluated and also the association with thyroid cancer risk, or other diseases risk to be studied. 

With the purpose of confirming this idea, we performed a case–control study to investigate the potential role of nETE dietary exposure in the high incidence of thyroid cancer in French Polynesia by assessing nETE pollution in locally produced food, then by estimating dietary intake of Polynesian population and studying its correlation with nETE fingernail concentrations among the natives of this Pacific island and finally the association of nETE dietary intake and fingernail concentrations with thyroid cancer risk and correlation with nETE nail concentrations.

## Materials and Methods

*Case-control study*

A detailed description of the methodology and the conduct of the case-control study has been published elsewhere (De Vathaire et al, 2010).

All patients diagnosed with a differentiated thyroid (follicular or papillary) cancer before the age of 56 between 1979 and 2003, born and living in French Polynesia, were eligible for the study. The cases were identified from the cancer registry of French Polynesia and medical insurance files and by the 4 endocrinologists in Tahiti. Of the 255 eligible thyroid cancer cases, 26 cases (10%) were not interviewed because:

They had died (n=14), could not be located (n=6), refused to participate (n=5) or were too ill to be interviewed (n=1). The final study population consisted of 229 cases (95% of the living potential cases).

For each eligible case, two potential controls closest in terms of the date of birth (±20 days for the first interviewed controls which changed to ±3 months afterwards) were randomly selected from the registry of births, which records all inhabitants born in French Polynesia, and matched by sex. 

Of the 458 randomly selected controls, 85 (19%) were not interviewed because: the subjects had died (n = 9), could not be located (n = 29), refused to participate (n = 29), were too ill to be interviewed (n = 2), or had left French Polynesia (n = 16). 

In total, the study population included 229 interviewed cases and 373 interviewed controls, with 85 cases (37%) matched to one control and 144 cases (63%) matched to two controls. We further excluded 6 cases and 9 controls for missing data on fingernail nETE and 2 controls due to missing data on weight.

All contact addresses were obtained from the territorial medical insurance plan, which covers all inhabitants, regardless of their professional status. Interviews were conducted face-to-face by trained Polynesian interviewers and medical staff, between 2002 and 2004, using a structured questionnaire. Questionnaire items included ethnicity, education, smoking habits, lifetime weight changes, personal and family history of thyroid disease and cancer, places of residence, gynaecological and linked to reproduction factors, medical x-ray exposure, and diet.

This questionnaire was adapted from the validated questionnaire of the French part of the European Prospective Investigation into Cancer and Nutrition (EPIC), according to foods consumed in French Polynesia (Bricas et al., 2001) The dietary questionnaire was on the consumption (frequency and quantity) of 66 food items in the year before interview. Portion sizes were estimated with a photo booklet validated by the EPIC study (Lucas et al., 1995).

All participants provided fingernail clippings on the date of the interview. Clippings were stored in paper envelopes at room temperature before being transferred to the toxicology centre of the “Institut National de Santé Publique du Québec” (INSPQ).

*Measures in nails*

Nail samples were digested under acidic conditions using concentrated nitric acid. The digest was then diluted and analysed by ICP-MS (inductively coupled plasma mass spectrometry) using an Elan DRC II system from PerkinElmer with auto sampler ESI-SC-4 and work station Elan version 3.0 (Dressler et al., 1998). The analytical method was fully developed and validated for monitoring purposes following 17025 ISO guidelines quality control (QC) was ensured by analysing three certified reference materials: GBW 09101, GBW 70601 and NIST 8415QC was run after calibration, after every 10th sample and at the end of each analytical sequence.

*Local food sampling*

The following locally produced foods or food groups were collected in each archipelago of French Polynesia: tubers (cassava, sweet potato, ufi: yam, taro, tarua), uru (breadfruit), cabbage, vegetables (long beans, fafa: Tahitian spinach), fê’i (orange banana), fruits (banana, mango, pawpaw, guava, passion fruit, watermelon, pineapple, copra), coconut water, ocean fish, lagoon fish, and shellfish (giant clam, other shellfish). This food sampling was carried out between 2011 and 2013 as a routine measurement since the nuclear tests in French Polynesia performed by the French Institute for Radiological Protection and Nuclear Safety (Institut de radioprotection et de sûreté nucléaire, IRSN) to follow environmental contamination in seven areas: Tahiti and Maupiti (Society Islands), Hao and Rangiroa (Tuamotu Archipelago), Mangareva (Gambier Islands or Mangareva Islands), Tubuai (Austral Islands) and Hiva Oa (Marquesas Islands). Foods were selected to cover the typical diet of the French Polynesian population. French Polynesia comprises 118 main islands (76 inhabited). It is composed of five archipelagos: The Windward Islands (Tahiti and Moorea) and the Leeward Islands (which make up the Society Islands), the Tuamotu atolls, the Gambier Islands, the Austral Islands, and the Marquesas Islands. Each of the composite samples from the seven areas of sampling was composed of up to five sub-samples from five different locations. Sampled fish species were Epinephelus microdon (Lagoon Fish), Naso brachycentron (Lagoon Fish), Thunnus orientalis (Sea fish), Thunnus alalunga (Sea fish), Thunnus albacares (Sea fish), Katsuwonus pelamis (Sea fish) and Coryphaena hippurus (Sea fish). In total, 124 samples were collected.

*Measures in local food*

Samples were lyophilised. Trace element measurements were carried out by the French national agency for food, environmental and occupational health safety (ANSES). Only Hg, Pb, Cd and As measurements were analysed in the present study, information on iodine and selenium daily intake used in the study has already been published elsewhere (Ren et al, 2014, Leufroy et al, 2015). Samples were prepared by microwave digestion in a closed-vessel and the ICP-MS was used for the detection and determination of trace and major elements levels in the different types of food samples (Chevallier et al, 2015). We considered total nETE concentrations, without any speciation analysis.

*Statistical methods*

Descriptive statistics and trend tests in univariate analysis were performed using non-parametric Wilcoxon rank test for association between a qualitative variable with two classes and a quantitative variable; and non-parametric Kruskal-Wallis rank tests for association between a qualitative variable with more than two classes and a quantitative variable.

The association between dietary exposure, nETE in nails and thyroid cancer risk and other parameters was investigated using generalised models. We used conditional logistic regression to estimate OR and 95% CI for thyroid cancer by dietary exposure and also by fingernail nETE concentrations. Trace elements dietary intake and fingernail concentrations were categorised into quartiles.

To study the association between dietary exposure and thyroid cancer risk, all models were conditioned on year of birth (<1945, 1945-1955, 1955-1965, ≥1965) and sex to reduce the potential bias, and then adjusted for year of birth (continuous, per year) to control for residual confounding by age. 

Multivariable models were additionally adjusted for factors known to be linked to thyroid cancer risk in French Polynesia (Xhaard et al., 2014) which are: ethnicity (Polynesian, mixed, other), education (primary school or lower versus middle school or higher), body mass index (BMI) at the moment of cancer (<25, 25-29.9, ≥30 kg/m2, missing), current smoking (yes versus no), radiotherapy treatment to head or neck (ever versus never), number of full term pregnancies, first-degree relative thyroid cancer history, iodine daily intake, selenium daily intake and energy intake.

These associations (thyroid cancer risk, nETE dietary intake and fingernail contents) were also stratified by age, gender and evaluated by smoking status, ethnicity, education level, BMI, age of diagnosis and radiotherapy treatment to the head and neck and dietary selenium and dietary iodine categories.

Possible modifications of the relation between nETE intake estimates and fingernail measurements, and thyroid cancer risk, by sex, smoking status, BMI, thyroid cancer histology, tumour size, number of full term pregnancies, first-degree relative thyroid cancer history, dietary iodine and dietary selenium, were evaluated using likelihood ratio test comparing a model with and without interaction terms. 

The study of correlation between nETE daily intake and pollutant fingernail concentrations was performed using Pearson product-moment correlation coefficient.

All statistical analyses were conducted using SAS software (version 9.4, SAS Institute Inc, Cary, NC, USA).

## Results

*Measures in food sampling*

*Mercury*

**Figure 1:**
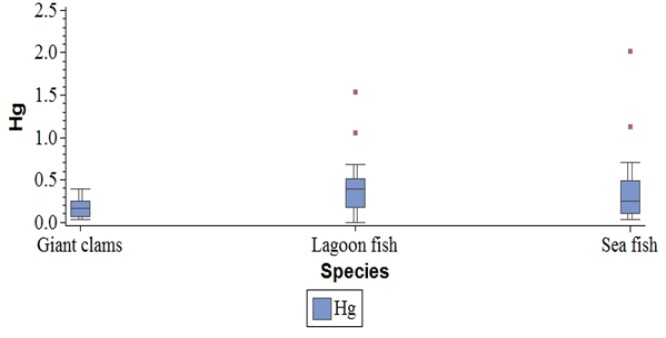
Distribution of Mercury in µg/g by Sea Products Types

Hg was essentially detected in fisheries (lagoon fish, sea fish and giant clams). Only two vegetable samples contained Hg: Fafa (Tahitian spinach) and coconut water, in one sample out of three for the first and one sample out of six for the second. Within fisheries, Hg was not detected only in Naso (lagoon fish species) in two samples from different geographic origins (Hao and Mangareva). Table 1 summarises the average Hg distribution in all samples of sea products. When taking into account archipelago and season, no significant difference was observed in Hg concentration between giant clams or fishes (p=0.3), nor among fishes between the offshore and the lagoon areas (p=0.3). Although based on 2 measures in Tahiti, median Hg concentration in giant clams was 0.035 µg/g (range: 0.03 to 0.04), compared to 0.23 (range: 0.07 to 0.39) in the other islands or atolls (p=0.04). Such a significant geographic variation was not observed for lagoon fishes, nor for offshore fishes. Figure 1 describes the distribution of Hg by sea products types.

*Lead*

**Figure 2:**
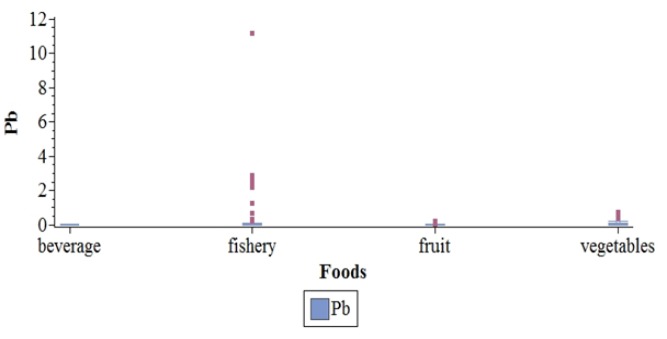
Distribution of Lead in µg/g by Food Types

In contrast to Hg results, Pb was present in almost all samples (110 samples from 124), in at least 2 samples of each aliment, and the most contaminated fish species was Naso. Figure 2 presents distribution of Pb by food types. No significant geographic variation was found in water, vegetables or fruits tested Furthermore, in fisheries, no significant difference was observed in Pb concentration among fishes between the offshore and the lagoon areas. 

However, in giant clams from Tahiti, this concentration was significantly lower than in other archipelagos (p= 0.04). Giant claims fished between December to February contained more Pb than those fished during other seasons (p=0.02). This fact was also seen in lagoon fishes caught in the same period (p=0.05).

*Cadmium*

**Figure 3:**
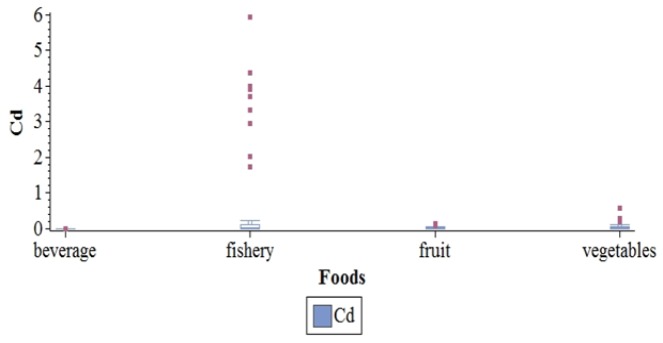
Distribution of Cadmium in µg/g by Food Types

Cd distribution by food types is presented by Figure 3. It was not detected in drinking water samples, although it was present in all other tested meal samples (117 samples from 124). Starchy food Cd concentrations were significantly higher in the Leeward Islands than in other archipelagos (p=0.04) in contrast to Tahiti, where this concentration was the lowest (p=0.03). In green vegetables, Cd concentration was higher in those produced during June to September (p=0.034). Cd concentration was also higher in offshore fishes than in lagoon ones (p=0.004). There was no difference between giant clams from different archipelagos or seasons.

*Arsenic*

**Figure 4:**
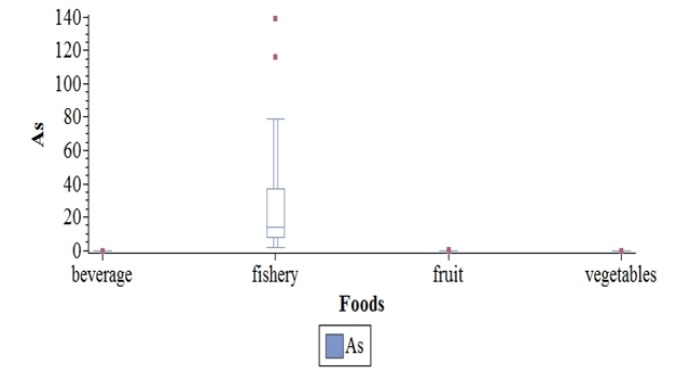
Distribution of Arsenic in µg/g by Food Types

As was detectable in at least one sampling in any species of vegetable and fruit, but in only 2 samples of drinking water. All sea products samples contained this metalloid. As concentration was significantly higher in fruits from atolls (p=0.02). Such a difference was also remarkable among giant clams, As concentration was also significantly higher for giant in or in atolls and in other islands than Tahiti (p=0.04). Lastly, As concentration was higher in offshore fishes fished between March and May than in others (p=0.02). As distribution by food types is presented in Figure 4. 

*Estimation of daily dietary exposure*

Geographic concentration differences were considered only for Hg in giant clams: two mean values were considered, depending on whether the geographic origins were Tahiti or not. We did not consider these differences for the other nETE since no wide variation was observed between different regions. Water measurements were not considered in this step due to their low number and important variability. 

**Table 1:** Mercury Distribution by Sea Products Type in µg/g

Nature	Mean (µg/g)	Standard deviation	Minimum	Maximum
Giant clams	0.18	0.13	0.03	0.39
Lagoon fish	0.43	0.39	<LOD	1.53
Sea fish	0.38	0.43	0.035	2.02

**Table 2:** Estimated nETE Daily Intake in µg/kg Body Weight among Cases and Controls

nETE daily intake in µg/ kg bw	Cases (n= 229)			Controls (n=371)		
	Mean (95%CI)	Median (25th-75thPercentiles)	Minimum- maximum values	Mean (95% CI)	Median (25th-75thPercentiles)	Minimum- maximum values
Hg	0.08 (0.07-0.09)	0.05 (0.03-0.10)	LOD-0.55	0.10 (0.09-0.12)	0.07 (0.04-0.13)	0-1.51
Pb	0.14 (0.11-0.16)	0.09 (0.04-0.15)	0.01-1.51	0.17 (0.16-0.19)	0.12 (0.21-0.07)	0.0007-1.69
Cd	0.07 (0.06-0.09)	0.04 (0.02-0.09)	0.002-0.69	0.12 (0.10-0.13)	0.07 (0.03-0.14)	0.0004-1.60
As	5.08 (1.54-6.65)	3.16 (1.54-6.65)	0.007-29.11	6.53 (5.85-7.20)	4.57 (2.45-8.19)	0.0001-73.53

**Table 3:** Odds Ratio of Thyroid Cancer Risk According to Estimated Daily Ingested Quantity of nETE in µg Reported by kg Body Weight

MTE daily intake per Quartile					
	Cases	Controls	Crude OR (95%CI)	Adjusted OR a (95%CI) per Quartile of daily intake (in µg/kg bw)	p for trend
Hg					
Q1 (0.00-0.039)	62	90	1*	1*	0.63
Q2 (0.039-0.07)	48	102	0.76 (0.46-1.17)	0.99 (0.56-1.76)	
Q3 (0.07-0.13)	30	119	0.58 (0.36-0.94)	0.63 (0.31-1.28)	
Q4 (0.13-1.51)	37	113	0.36 (0.42-1.092)	0.97 (0.41-2.29)	
Pb					
Q1 (0.0007-0.06)	65	88	1*	1*	0.1
Q2 (0.06-0.11)	46	103	1.024 (0.65-1.60)	0.68 (0.39-1.20)	
Q3 (0.11-0.19)	30	120	0.61 (0.37-0.99)	0.39 (0.20-0.75)	
Q4 (0.19-1.69)	36	113	0.66 (0.39- 1.095)	0.72 (0.35-1.49)	
Cd					
Q1 (0.0004-0.03)	68	85	1*	1*	0.26
Q2 (0.034-0.06)	43	106	0.64 (0.38-0.94)	0.38 (0.20-0.73)	
Q3 (0.06-0.12)	35	115	0.61 (0.39-0.95)	0.33 (0.16-0.97)	
Q4 (0.12-1.6)	31	118	0.57 (0.36-0.91)	0.28 (0.12-0.68)	
As					
Q1(0.0001-2.11)	59	93	1*	1*	0.87
Q2 (2.11- 3.99)	48	102	0.78 (0.49- 1.22)	1.19 (0.65-2.19)	
Q3 (3.99-7.84)	32	117	0.50 (0.30- 0.82)	0.87 (0.44-1.61)	
Q4 (7.84-73.53)	38	112	0.75 (0.45-1.20)	1.28 (0.58-2.81)	

^a^, Stratified by age, gender, and adjusted for smoking status, ethnicity, education level, number of full term pregnancies, BMI, radiotherapy for previous cancer, first-degree relative thyroid cancer history Iodine daily intake, Selenium daily intake and energy intake; * Subgroup taken as reference.

**Table 4:** Statistically Significant Interaction Factors in Relation to Thyroid Cancer Risk

		MTE	Thyroid cancer risk		
			Adjusted OR per Quartile of daily intake (in µg/kg bw)	p value	p interaction
Pregnancies number (among women)	<3	Pb	0.44 (0.27-0.73)^a^	<0.0005	0.005
	>=3		0.98 (0.72-1.35)^a^	0.9	
History of cancer in first degree relatives	No	Pb	0.67 (0.52-0.88)^b^	<0.005	0.01
	Yes		2.88 (0.85-10.7)^b^	0.07	
	No	Hg	0.82 (0.62-1.08)^b^	0.1	0.06
	Yes		1.75 (0.62-4.9)^b^	0.2	
	No	As	1.91 (0.71-1.17)^b^	0.4	0.05
	Yes		2.48 (0.73-8.47)^b^	0.1	

^a^, Model stratified by age, gender, and adjusted for smoking status, ethnicity, education level, BMI, radiotherapy for previous cancer, first-degree relative thyroid cancer history Iodine daily intake, Selenium daily intake and energy intake; ^b^, Model stratified by age, gender, and adjusted for smoking status, ethnicity, education level, BMI, radiotherapy for previous cancer, pregnancies number Iodine daily intake, Selenium daily intake and energy intake.

**Table 5:** nETE Fingernail in µg/g among Cases and Controls

MTE in fingernail (µg/g)	Cases			Controls		
	Mean (95% CI)	Median (25^th^-75thPercentiles)	Minimum- maximum values	Mean (95%)	Median (25th-75thPercentiles)	Minimum- maximum values
Hg	1.10 (0.96-1.24)	0.92 (0.53-1.45)	<LOD -6.2	1.20 (0.90-1.30)	1 (1.07-1.53)	<LOD-45
Pb	1.12 (0.83-1.42)	0.63 (0.39-1.00)	<LOD -17	1.73 (0.95-2.50)	0.63 (0.39-1.10)	<LOD-140
Cd	0.04 (0.02-0.05)	<LOD (0-0)	<LOD-0.52	0.08 (0.03-0.12)	0 (0-0)	<LOD-7.6

**Table 6:** Odds Ratio of Thyroid Cancer Risk According to nETE Fingernail in µg /g of Nail

	Cases	Controls	Crude OR (95%CI)	Adjusted OR a (95%CI) per Quartile of nETE fingernails (in µg/g)	p for trend
Hg					
Q1 (0.00-0.62)	57	104	1*	1*	0.7
Q2 (0.62-1.00)	38	103	0.60 (0.38-1.03)	0.63 (0.32-1.20)	
Q3 (1.00-1.50)	38	101	0.68 (0.41-1.13)	0.69 (0.35-1.34)	
Q4 (1.50-45)	44	106	0.63 (0.37-1.07)	0.88 (0.42-1.81)	
Pb					0.4
Q1 (0.00-0.39)	47	110	1*	1*	
Q2 (0.39-0.63)	41	106	0.82 (0.47-1.43)	0.94 (0.46-1.93)	
Q3 (0.63-1.10)	48	97	1.02 (0.58-1.77)	1.19 (0.58-2.42)	
Q4 (1.10-140)	41	111	0.79 (0.47-1.34)	0.68 (0.34-1.36)	
Cd					0.3
Q1 (0.00)	139	332	1*	1*	
Q2 (0.00-0.16)	20	42	0.90 (0.45-1.78)	0.85 (0.35-2.07)	
Q3 (0.16-7.60)	18	50	0.75 (0.39-1.43)	0.69 (0.31-1.52)	

^a,^ Stratified by age, gender, and adjusted for smoking status, ethnicity, education level, BMI, age of diagnosis and radiotherapy treatment to the head and neck and dietary selenium and dietary iodine categories; *, Subgroup taken as reference.

**Table 7:** Statistically Significant Interaction Factors in Relation to Thyroid Cancer Risk

			Thyroid cancer risk		
			OR ^a^ (95% CI)	p value	p interaction
BMI class	Low	Cd fingernail in µg/g	0.70 (0.5-1.00)	0.05	0.07
	High		1.05 (0.72-1.51)	0.7	

^a^, Model stratified by age, gender, and adjusted for smoking status, ethnicity, education level, BMI, age of diagnosis and radiotherapy treatment to the head and neck and dietary selenium and dietary iodine categories.

*Mercury*

Table 2 summarises the daily Hg intake according case-control status. However, the average daily intake of total Hg among all participants was 0.10 µg/kg bw/day, and the maximum value was 1.51 µg/kg bw/day. Only two participants were above the recommended daily intake limits of total Hg (0.60 µg/kg bw/day according to the last JECFA value (WHO, 2011)) However, all Hg dietary intake came from fish and shellfish, which mostly contain methyl Hg; compared to the WHO maximum limit (0.22 µg/kg bw/day (WHO, 2011)), 53 persons were above this, and according to the new value established by EFSA (0.18 µg/kg bw/day (Panel, 2011)), 87 participants exceeded this limit. 

Polynesians had higher exposure than other ethnicities. Nonetheless, “Windward Islands” inhabitants had the highest Hg exposure compared to other archipelagos. A description of Hg dietary intake from different tested food types is presented in supplemental Table 1.

*Lead*

Pb dietary intake by food types among cases and controls is summarized in supplemental table 2. The average daily intake of Pb among all participants was 0.16 µg/kg bw/day; the maximum value was 1.69 µg /kg bw/day while the minimum was 0.07 10-2 µg/kg bw/day. All participating subjects were below the JECFA recommended daily intake limits (3 µg/kg bw/day (WHO, 2011)) and even compared to the EFSA value, which is lower (1.5 µg/kg bw/day (Alexander, 2010)), only 2 participants were above. Pb exposure was higher in women than in men (p=0.040), among “Australes Islands” inhabitants than in other archipelagos inhabitants and in Polynesians than in other ethnicities. The exposure according case-control status is described in Table 2.

*Cadmium*

The average daily intake of Cd was 0.10 µg/kg bw/day and the maximum value was 1.6 µg/kg bw/day, while the minimum was 0.04 10-2 µg/kg bw/day among all participants. In total, 45 persons of the studied population were above the recommended daily intake limits (0.8 µg/kg bw/day according to the last JECFA value (WHO, 2011)), but compared to the EFSA value, which is 0.35 µg/kg bw/day ( Alexander et al, 2009), this limit was exceeded by 63 participants. Polynesians had higher exposure than other ethnicities (p=0.0032). The table 2 summarise the Cd exposure values among cases and controls. The daily intake of Cd from different food types among cases and controls is presented in supplemental Table 3.

*Arsenic*

The As daily intake by food type is presented in supplemental table 4. The average daily intake of As among cases and controls was 6.10 µg/kg bw/day; the maximum value was 73.53 µg/kg bw/day while the minimum was 0.0001 µg/kg bw/day. In the present study, all analyses were carried out for total (organic and inorganic) As. However, it is well-known that most As found in fish and shellfish is organic As, which is the less toxic form. The percentage of inorganic As in sea products has been reported to be less than 11% (Muñoz et al., 2000). Taking this into account, in the present study, the intake of As was below the WHO recommended daily intake limits for total As dietary exposure (2.1 µg/kg bw/day (WHO, 2011)), whereas the maximum acceptable daily load for organo-arsenicals from fish and shellfish consumption, published by the WHO in 1989, is 50 µg/kg bw/day (WHO, 1993). 

Polynesians had higher exposure than other ethnicities (p=0.00043) “Tuamotu” archipelago inhabitants had the highest As exposure level. Values among cases and controls are presented in Table 2.

*Thyroid cancer hazards in relation to contamination*

*Univariate analysis*

Compared to lower quartiles, higher exposure was associated with a lower thyroid cancer risk for all nETE (Table 3).

*Multivariate analysis*

The multivariate model was stratified by age and gender, and adjusted for smoking status, ethnicity, education level, BMI, age of diagnosis, radiotherapy treatment to the head and neck and selenium and iodine daily intake. Cd daily intake was inversely associated with thyroid cancer risk (Table 3).

*Interactions*

Each µg/day/kg bw of Pb increased the thyroid cancer risk by more than 4 times in patients with a history of cancer in first-degree relatives than in those without (p for interaction=0.01), and 2 times higher in women with more than 3 pregnancies than in those with none or less than 3 (p for interaction =0.005)

For As exposure, each µg/day/kg bw increased thyroid cancer risk by 30% more in patients with a history of cancer in first-degree relatives than in those without (p for interaction=0.05). The interaction between history of cancer in first-degree relatives and Hg exposure was found for the limit of significance (p for interaction=0.06). Thyroid cancer risks doubled among patients with a history of cancer in first-degree relatives by each µg/day/kg of Hg intake (Table 4).

*Measures in nails*

Table 2 describes the nETE fingernail concentrations according case-control status. Detailed results for the total population are summarised below: 

*Mercury*

Among the studied population, the mean Hg fingernail concentration was 1.2 µg/g. The value among the whole population was above the average in 34% (n=199) of the nail samples and was below LOD in 32 others. Highest levels were found in older people than in younger individuals (p=0.01), in Windward archipelago inhabitants than others archipelago inhabitants (p=0.02), and in men than in women (p=0.01). However, the lowest Hg fingernail concentrations were observed in Polynesians (p=0.001) compared to other ethnicities and among people with a low education level (p<0.0001). 

*Lead*

In 15% (n=88) of fingernail samples, Pb concentration was higher than the observed average of 1.55 µg/g and below the LOD in 42 samples. This concentration was higher in men than in women (p<0.0001) and among Tuamotu-Gambier archipelago inhabitants (p=0.013) and those with a low education level (p=0.039). However, no effect of the smoking status, age or ethnicity was observed. 

*Cadmium*

Mean Cd fingernail concentration was 0.069 µg/g, this value was exceeded in 22% (n=130) and below the LOD in 75% (n=455) of the samples. This concentration was higher in men than in women (p=0.0009) and in Tuamotu-Gambier archipelago inhabitants (p=0.023). However, there is no observed effect of age, education level, smoking status or ethnicity on Cd fingernail concentration.

*Correlation between nails and dietary exposure*

Among the studied nETE, no significant correlations were found between estimated daily intakes and fingernail concentrations, except for Cd; a positive correlation was observed between daily exposure and male gender, as Cd fingernail values were higher among male participants than in women for the same daily exposure (r =0.22, p=0.01). 

*Hazards in relation to nails.*

*Univariate analysis*

No significant association was found between the different levels of fingernail nETE and thyroid cancer risk in univariate analysis (Table 6).

*Multivariate analysis*

The multivariate model was stratified by age and gender, and adjusted for smoking status, ethnicity, education level, BMI, age of diagnosis and radiotherapy treatment to the head and neck, but no significant association was found between fingernail nETE and thyroid cancer risk in multivariate analysis (Table 6).

*Interactions*

Interactions were tested with gender, BMI, smoking status, number of pregnancies, iodine fingernail and selenium fingernail. Thyroid cancer risk was decreased of 30% by each µg of Cd fingernail among participants with lower BMI class (below 25 kg/m^2^) than others with a higher BMI (p=0.07) (Table 7). However, no significant interactions were found with gender, smoking status, number of pregnancies, iodine fingernail and selenium fingernail.

## Discussion

The main purpose of the present study was to provide quantitative information on food contamination by Hg, Pb, As and Cd in French Polynesia and its potential correlation with measurements taken in fingernails of natives of this overseas territory. We also investigated the potential association between these nETE and differentiated thyroid cancer risk.

*Mercury*

Most of the detected Hg in samples came from sea products, which are known to be a source of methylmercury (MeHg) ( Holmes et al., 2009; Olmedo et al., 2013). Different mechanisms of MeHg toxicity are discussed, like oxidative stress and the inhibition of macromolecule synthesis (Soldin et al., 2008).The role of Hg as an endocrine disruptor affecting thyroid hormones analysed and found to be is inversely associated with thyroid hormone blood levels by several studies (Soldin et al., 2008; Ursinyova et al., 2012; Chen et al., 2013).

It was also suspected to be a promoting factor for thyroid cancer (Zaichick et al., 1995, Malandrino et al., 2016). Hg interacts with selenium, which has been considered by several studies to be a protective element against MeHg poisoning (Ralston et al., 2007); this finding is important because, most of the Hg dietary intake came from sea products, which are also the main source of dietary Se. Nevertheless, in the present study, we failed to show an interaction between Se and Hg estimated dietary intake and thyroid cancer risk. A stable ratio between Hg and Se intake could have a protective role against Hg toxicity (Ralston et al., 2007; Soldin et al., 2008), which could partially explain those results; nevertheless, the risk/benefice balance of fish consuming still remains controversial (Olmedo et al., 2013). In addition, some dietary studies have shown that fruit consumption has a protective role against Hg exposure (Passos et al., 2007).

In the present study, the mean value of Hg fingernails was 1.2 µg/g in nails (standard deviation=2.0), close to the value of 1.3 µg/g (standard deviation= 1.4) in Japanese populations by Suzuki et al., (1989). But the present study failed to demonstrate a correlation between nETE fingernail and their dietary intake and also to show a relation between nETE fingernail levels and differentiated thyroid risk.

however, this correlation was evidenced by Rees et al, with Hg toenail being strongly correlated with finfish and shellfish consumption (Rees et al., 2007), but not to age, or education level. In contrast, in our results, Hg fingernail was influenced by age and education level. This is consistent with an American study that included data from 4,344 participants and showed that age, gender, ethnicity, alcohol, education level and fish consumption consistently predict nail Hg levels (Xun et al., 2013). Polynesians had the lower Hg fingernail concentration; however, they were the most exposed. This could be explained by the effect of genetic polymorphism on Hg levels in the blood and hair, as suggested by other studies (Wang et al., 2012; Barcelos et al., 2013; Barcelos et al., 2015; Parajuli et al., 2016 ). 

*Lead*

Pb was detectable in almost all studied food samples, it is known to be an endocrine disruptor affecting thyroid hormone homeostasis by decreasing blood levels (Kahn et al., 2014). The present study suggests that Pb exposure could increase the thyroid cancer risk among women with a higher number of pregnancies more than in women with a lower number of pregnancies or none. Such a result concurs with the results of two other studies where a positive association between thyroid cancer risk and the high number of pregnancies had been evidenced (Truong et al., 2005; Leux et al., 2012). An increased Pb bone turnover has been detected in lactating and menopausal women and also during and after pregnancy, which means that endogenous Pb sources from past exposures influence blood Pb levels (Burger et al., 2003; Rothenberg et al., 2000, Gochfeld et al., 1997). This increase in blood Pb could explain the positive association of pregnancy number to a higher thyroid cancer risk. 

Besides, there are more dietary factors controlling blood Pb in women, like calcium intake, which is suspected to decrease blood Pb (Kostial et al., 1991). Pb exposure was found to increase thyroid cancer risk more in participants with a history of cancer in first degree relatives than in persons without. Pb was the most frequent pollutant detected in fingernail samples. The validity of fingernails as a Pb biomarker is often discussed since it reflects only the exposure during last months and also the lack of reproducibility descripted by Gulson (1996) in his study. Pb levels measured in the same fingernails and toenails of various subjects are very variable. The same result was also presented by Trunova et al., (2009), their results found that the contents of the chemical elements, Pb among others, in donor nails change with time. Such results showed that nail specimens offer only limited scope in Pb exposure assessing.

*Cadmium*

Cd blood concentration has been associated with later stages of thyroid cancer and higher thyroid tissue levels of Cd are found in multifocal patients (Chung et al., 2016). It has also be found associated with thyroid hormone homeostasis disturbance (Chen et al., 2013; Buha et al., 2013; Wade et al., 2002). Among the studied nETE, Cd seems to be the best candidate for an association with thyroid cancer (Liu et al., 2007; Chung et al., 2016; Malandrino et al., 2016). Nonetheless, this relation could not be demonstrated in the present studyMost participants were not exposed to high Cd levels, and many other elements and nutrients are supposed to have an antagonistic effect on Cd-induced thyroid dysfunction, like Se (Chung et al., 2016), zinc (Hammouda et al., 2008) and ascorbic acid (Gupta et al., 1998). Therefore, those nutrients reduce cancer risk. The negative association we evidenced between Cd consumption and thyroid cancer risk could be due to a common origin: sea products consumption is the main source of both dietary selenium and Cd (Kaneko et al., 2007; Dewailly et al., 2008).

Cd was the less frequently reported nETE in fingernail samples. The correlation found between Cd fingernail and gender concurs with the results of other studies assessing the validity of the nails as a biomarker and showing that the concentration of Cd in fingernails varies individually according to gender and other different factors (Kim et al., 2011).

*Arsenic*

A case reported by Au et al in 2014 evidenced a higher concentration of As in the thyroid carcinoma tissue in a patient treated 10 years ago by oral arsenic-trioxide therapy compared to 4 controls treated for thyroid carcinoma. This suggests a long-term higher thyroid cancer risk, after As oral intake. As is a disrupting factor of thyroid hormone homeostasis; the analysed data from the American National Health and Nutrition Examination Survey demonstrated a negative association between As biological levels and thyroid hormones (Jain et al., 2016). Another study concluded, by comparing rural and urban habitants, to a significantly higher levels of thyroid-stimulating hormones (TSH) and thyroglobulin (TGN) and lower free thyroxin (FT4) and free triiodothyronine (FT3) in participants with higher As exposure (Ciarrocca et al., 2012).

The assessed exposure in those studies came from different sources: dietary, air pollution and water. However, in the present study, only locally produced foods were assessed rather than drinking water. Even inorganic As (iAs) is the most toxic species (ATSDR, 2007); this metalloid was only dosed as total As. The principal source of As intake in our study was sea food. As intake could be considered less toxic since fish and shellfish As is mostly found in an organic form (about 90% of fish As) (Muñoz et al., 2000; Navas-Acien et al., 2011; Ng et al., 2011). Considering those facts, in the current study, the intake of As would not be of concern for participants.

*Study limitations*

As a whole, locally produced foods were not the cause of an overexposure to Hg (and methyl Hg), Pb and As. 

The present study did not confirm or suggest an association between estimated daily nETE intake and thyroid cancer risk, nor a difference between nETE fingernail concentrations of cases and controls. Some uncertain sources exist and influence those results like the limited number of food samples and also the nature of chosen biomarkers: fingernails. Fingernail is validated by The French Institute for Public Health Surveillance for only Hg bio monitoring (Fréry et al., 2011).

The dietary exposure estimation was built on food samples measurements; however, their number was limited compared to the kinds of food studied and despite sampling covering a large geographical area and a big number of species. Besides, since the participants answered the food questionnaire after being diagnosed with a thyroid cancer risk, recall bias could be possible. 

Even if several recent studies analysed the use of nails as biomarkers of different minerals and nETE and to relate their concentration to some diseases risk such as amyotrophic lateral sclerosis, children obesity, hearing loss and acute promyelocytic leukaemia (Karatela et al., 2018; Chen et al., 2018; Li et al., 2018; Xu et al., 2018; Andrew et al., 2018; White et al., 2018). In the present study, no relation was found by between nETE fingernail, dietary exposure and thyroid cancer risk. Fingernail sampling was performed in post-diagnosis, but this delay has little chance of affecting our results since it has been noted that over a period of six years, some nail trace elements like Hg had good reproducibility (Garland et al., 1993). Accordingly, our results are probably not due to the fact that we investigated post-diagnosis trace element levels rather than pre-diagnosis ones.

A major limitation of the present study came from the fact that nETE were measured on traditional Polynesian locally produced food (mostly fruits, vegetables, and sea products). This was done because other food kinds consumed in French Polynesia have a lot of origins, New Zealand, Australia, Hawaii, but also, and mostly, France and rest of the world. Nevertheless, sampled food accounted for only about 25% of the total daily energy intake.

Another main limitation of our study is the small number of samplings per type of aliment, which prevents us from performing a detailed analysis of the source of variability of pollutant concentrations in each of type. Consequently, we considered geographical variation only for Hg in fishes and giant clams: Tahiti versus other islands. The magnitude of the variability we observed in pollutant concentration for any type of aliments shows that more measurements are necessary to correctly investigate this. 

According to our results, no association was found between estimated pollutant intake and thyroid cancer risk, except for Cd, which was found to be associated with a lower risk. This result is the opposite of other studies findings and could be explained by the high presence of both Se and Cd in sea products and the antagonist effect of Se on Cd and Hg-induced alterations. However, this possible relationship needs more investigation.

A possible role for fish consumption could be discussed since it is also an important source of selenium, iodine and other elements which could reduce thyroid cancer risk (Köhrle et al., 1999; Cléro et al., 2012; Luque-Garcia et al., 2013).

Several nutrients are involved in thyroid function and thyroid cancer risk; copper and zinc could have a role in maintaining the balance of thyroid hormones (Hammouda et al., 2008; Przybylik-Mazurek et al., 2011). 

Regular intake of multivitamins could play the same role (Mack et al., 2002). Dietary nitrate and nitrite are also positively associated with thyroid cancer risk (Ward et al., 2010; Kilfoy et al., 2011; Choi et al., 2014). 

Besides, the present study did not take into account the effect of other dietary factors suspected to be protective against thyroid cancer, like alcohol (Sen et a.l, 2015; Mack et al., 2002; Allen et al., 2009). 

In Conclusion the present study showed that, overall, locally produced food in French Polynesia are not a source of nETE over contamination. The results failed to evidence an association between nETE daily intake and thyroid cancer risk. nETE fingernail contents were not informative about dietary exposure and not associated to thyroid cancer risk. Due to the complexity of thyroid function and its regulation, establishing a potential relationship between dietary exposure to nETE and thyroid cancer risk requires more studies based on precise exposure assessments or nETE bioassays. Total environmental exposure should also be assessed since dietary daily intake it is not the only mechanism of nETE exposure.
